# Parallel Clustering Algorithm for Large-Scale Biological Data Sets

**DOI:** 10.1371/journal.pone.0091315

**Published:** 2014-04-04

**Authors:** Minchao Wang, Wu Zhang, Wang Ding, Dongbo Dai, Huiran Zhang, Hao Xie, Luonan Chen, Yike Guo, Jiang Xie

**Affiliations:** 1 School of Computer Engineering and Science, Shanghai University, Shanghai, P.R.China; 2 High Performance Computing Center, Shanghai University, Shanghai, P.R.China; 3 College of Stomatology, Wuhan University, Wuhan, P.R.China; 4 Key Laboratory of Systems Biology, Shanghai Institutes for Biological Sciences, Chinese Academy of Sciences, Shanghai, P.R.China; 5 Department of Computing, Imperial College London, London, United Kingdom; Semmelweis University, Hungary

## Abstract

**Backgrounds:**

Recent explosion of biological data brings a great challenge for the traditional clustering algorithms. With increasing scale of data sets, much larger memory and longer runtime are required for the cluster identification problems. The affinity propagation algorithm outperforms many other classical clustering algorithms and is widely applied into the biological researches. However, the time and space complexity become a great bottleneck when handling the large-scale data sets. Moreover, the similarity matrix, whose constructing procedure takes long runtime, is required before running the affinity propagation algorithm, since the algorithm clusters data sets based on the similarities between data pairs.

**Methods:**

Two types of parallel architectures are proposed in this paper to accelerate the similarity matrix constructing procedure and the affinity propagation algorithm. The memory-shared architecture is used to construct the similarity matrix, and the distributed system is taken for the affinity propagation algorithm, because of its large memory size and great computing capacity. An appropriate way of data partition and reduction is designed in our method, in order to minimize the global communication cost among processes.

**Result:**

A speedup of 100 is gained with 128 cores. The runtime is reduced from serval hours to a few seconds, which indicates that parallel algorithm is capable of handling large-scale data sets effectively. The parallel affinity propagation also achieves a good performance when clustering large-scale gene data (microarray) and detecting families in large protein superfamilies.

## Introduction

Data clustering is to group a set of objects in such a way that objects in the same group (cluster) have higher similarity with each other than those in the other groups (clusters). It is a common technology for data mining and analysis in many fields, such as pattern recognition, machine learning, bioinformatics and so on. Many novel clustering algorithms have been introduced to handle biological problems, including protein families/superfamilies detecting [1], [2], metabolic networks analysis [3]–[5] and protein-protein interaction (PPI) networks analysis [6], [7].

In the last decade, many clustering algorithms were proposed and widely applied to the biological researches [8]–[13] (see [14]–[16] for a comprehensive survey). One of the most successful clustering algorithms is the Markov Cluster algorithm (Tribe-MCL) [1]. In the original publication, it was used to detect the protein families in the protein-protein interaction networks based on the graph theory. The algorithm simulates the random walks within the graph by alternation of two operations, called the Expansion and the Inflation. Spectral clustering, was first introduced into the image processing [17]. It was recently applied to the protein sequence clustering problems [2]. Spectral clustering requires a long runtime, and the cluster number is required to be specified manually. In microbial community analysis, some classic clustering algorithms such as linkage, graph partition are taken to handle the difference between microbial sequences. The correlation between the comparison of the human microbiome and various disease can be extracted from the clustering results.

The rapid increment in biological data sets scale poses great challenges for sequential algorithms, and makes the parallel clustering algorithms more attractive [18]–[20]. Chen et al. implemented a parallel spectral clustering [18]; Ekanayake et al. applied the cloud technologies into the clustering [19]. Bustamam et al. designed the sparse data structure and implemented the sparse MCL algorithm on the GPU [20].

This paper focuses on the parallel biological clustering for large-scale data sets in the distributed system. A promising algorithm called Affinity Propagation [21] is considered in our work. In the biological researches, the affinity propagation algorithm has been widely used [22]–[26]. Affinity propagation algorithm has many advantages and outperforms some famous algorithms such as the k-means, spectral clustering and super-paramagnetic clustering [27]. Moreover it doesn't require a specific cluster number. Compared with the Tribe-MCL algorithm, the result of affinity propagation is less sensitive to its input parameter. However, its time and space complexity become a great bottleneck when handling large-scale data sets. Given a data set with 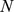 data points, the algorithm has to treat three 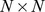 matrices. Two messages are computed iteratively when algorithm is running, and the time complexity of computing each message is about 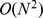. In order to address these challenges, we implemented our parallel affinity propagation algorithm in the distributed system. Distributed system can supply huge memory size and great computing capacity, so it is promising to design and implement large-scale biological applications on it. To our best knowledge, there are few works focusing on the parallel affinity propagation algorithm [28]–[32]. These works implemented the parallel affinity propagation algorithm on the memory-shared, GPU and MapReduce parallel architectures. The limitation on memory size and computing capacity of memory-shared parallel architecture make it difficult to handle large-scale data sets; While for the MapReduce parallel architecture, although it can supply the huge storage space, the runtime of parallel algorithm running on this architecture is much longer because of its running principle. The GPU parallel architecture has a great computing capacity, and its memory size also becomes larger in recent years. However, the GPU architecture is not good at doing the logic operations. Considering there are many logic operations in affinity propagation algorithms, we think that the GPU parallel architecture is not an appropriate parallel architecture. Compared with these parallel architectures, the distributed system is the most suitable for developing parallel affinity propagation. Compared with the works in publications [28], [29], our method optimizes the global communication, so the parallel algorithm runs faster. Our parallel AP algorithm is compiled in the CentOS System using the gcc-4.1.4 compiler. The version of MPI is 3.0.1. All source code is available on the website: http://hpca.shu.edu.cn/mpiap.

## Result and Conclusion

### Assessment of the Algorithm Running Performance

In order to measure the efficiency of the parallel clustering algorithm, the runtime and speedup of the algorithm are recorded. Two parts of runtime are measured. The first one is the runtime of constructing the similarity matrix, and the second is the runtime of the clustering algorithm. Our parallel clustering algorithm runs on the cluster computers environment. The cluster consists of 16 nodes, and in each node, there are 1 quad core Intel Xeon processor and 8 GB memory. All nodes are connected with each other by the gigabit Ethernet.

**Figure 1:**
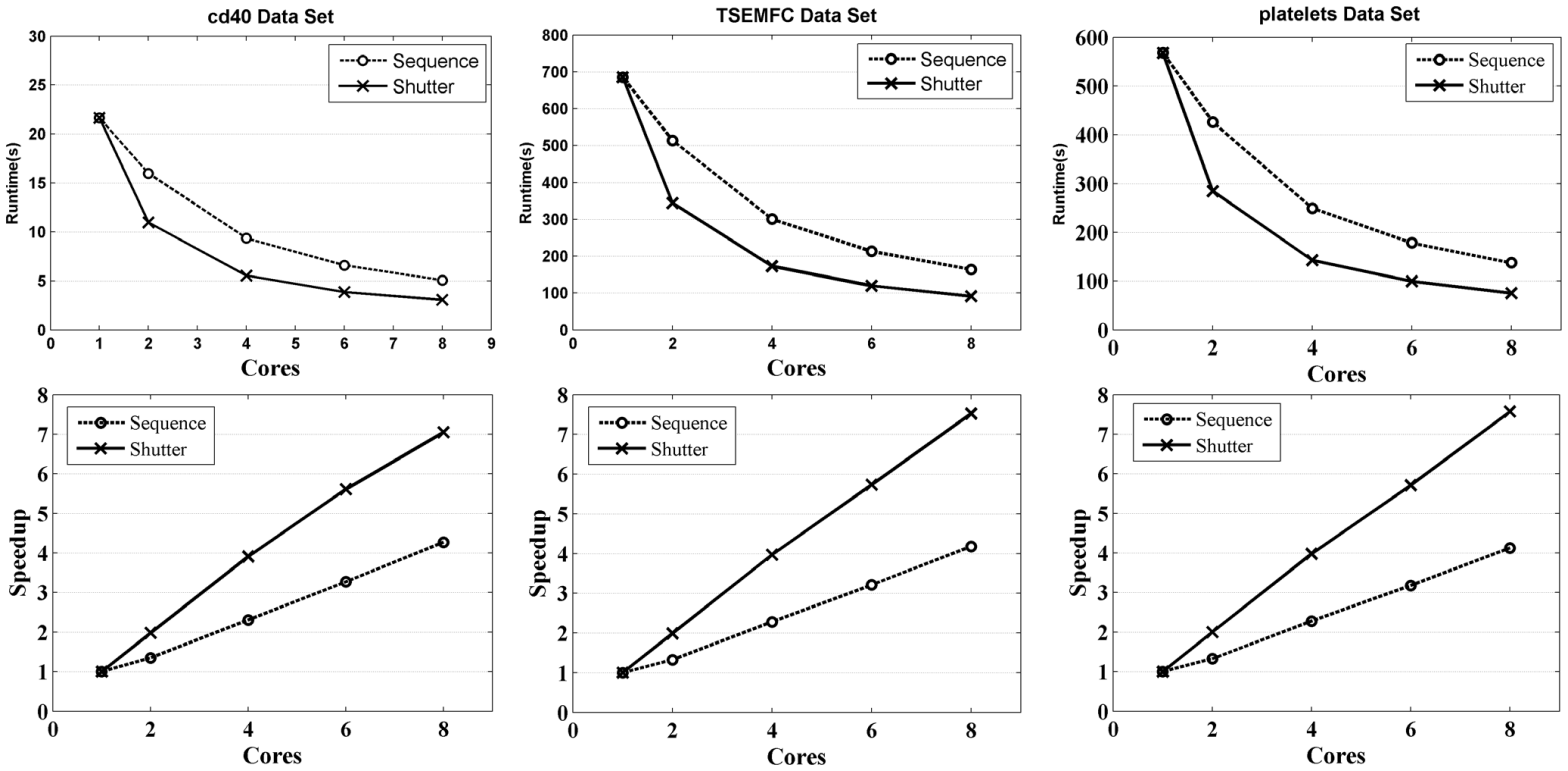
Runtime and speedup of constructing similarity matrix for different data partition ways. The 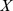 axis represents the number of cores, and the 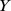 axis represents the runtime and speedup.

Five data sets (3 gene and 2 protein) were used in the experiments. Figure 1 gives the runtime and speedup (runtime of sequential algorithm over that of parallel algorithm) of two different ways of data partition for constructing the similarity matrix. The results shows that the shutter partition achieves much higher speedup than the sequence partition. Because the shutter partition makes the computing load more balanced, all cores can be fully utilized. Furthermore, because the memory-shared architecture is used, there are no communication cost. Table 1 lists the accelerating efficiency (speedup over the number of used cores).

**Table 1:** Comparison of accelerating efficiency for different data partition ways.

	Shutter Partition (%)				Sequence Partition (%)			
Data set	2 cores	4 cores	6 cores	8 cores	2 cores	4 cores	6 cores	8 cores
platelets	100	99.5	95.2	94.6	66.5	57.0	53.0	51.6
TSEMC	99.5	99.3	95.5	94.0	66.5	57.0	53.5	52.3
cd40	99.0	98.8	93.5	88.1	67.5	57.8	54.5	53.4

**Figure 2:**
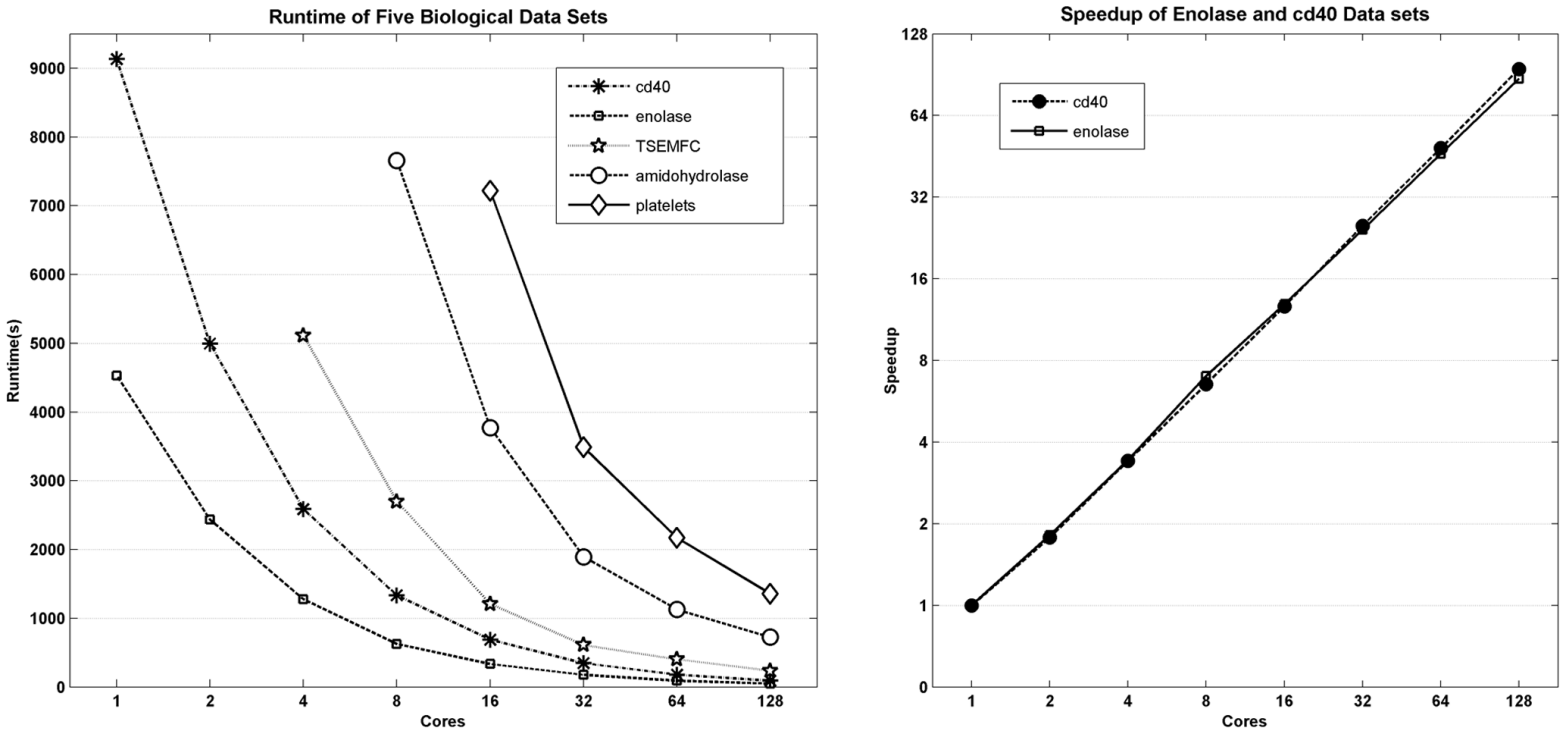
Runtime and speedup of parallel affinity propagation algorithm. (a) shows the runtime of the five biological data sets. (b) shows the speedup of cd40 and enolase data sets.

Table 2 and Figure 2(a) show the runtime of the parallel clustering algorithm. In Table 2, N/A represents the runtime of that data set is not available because of the limitation of the memory size. As the number of cores increase, the runtime of parallel affinity propagation decreases greatly. For cd40 and enolase data sets, the sequential algorithm runtime is obtained, so the speedup is calculated and shown in Figure 2(b). Compared with the sequential algorithm, the parallel algorithm achieves a promising speedup, so it can handle the large-scale biological data sets effectively.

**Table 2:** Runtime of the parallel affinity propagation algorithm.

	Runtime(sec)							
Data Sets	1 core	2 cores	4 cores	8 cores	16 cores	32 cores	64 cores	128 cores
cd40	9134.43	4995.28	2587.66	1334.10	687.59	346.21	178.57	91.05
TSEMFC	N/A	N/A	5113.52	2697.29	1357.79	711.86	404.08	236.88
platelets	N/A	N/A	N/A	N/A	7214.63	3489.62	2167.57	1357.54
Enolase	4531.59	2434.62	1277.32	627.66	335.98	176.79	92.66	48.70
Amidohydrolase	N/A	N/A	N/A	7660.48	3772.05	1894.40	1127.23	725.85

### Assessment of the Clustering Performance on Gene Data

For gene data, Gene Ontology Overlap Score is used to measure the clustering results. The Gene Ontology (GO) project provides an ontology of defined terms representing gene product properties. The ontology covers three domains: molecular function, biological process and cellular component [33]. In the GO Overlap measurement, the Jaccard and PR indices are introduced to score the clustering results [34].

**Jaccard:** given two sets, the Jaccard score is defined as the size of the intersection over the size of the union. For clusters 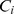 and 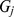, their Jaccard score is defined as 
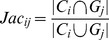
.

**PR:** The PR score includes two parts, the Precision score and the Recall score. For clusters 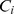 and 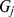, 
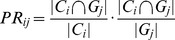
 denotes their PR score.

For each data set, we take an average score over all clusters, weighted by cluster size, to get the 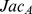 and 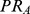 score. Also, we take an maximum score from all clusters, to get the 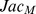 and 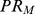 score. The Jaccard and PR scores range from 0 to 1, and the higher values indicate the better agreement of the uncovered clusters with functional modules corresponding to GO. The scores of three gene data sets are listed in TABLE 3.

**Table 3:** GO overlap analysis of high-throughput gene data sets.

	Molecular Function				Biological Process				Cellular Component			
Data Sets	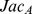 a	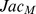 b	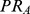 c	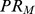 d	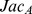	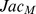	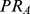	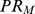	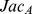	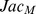	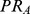	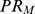
TSEMFC	0.133	0.167	0.065	0.082	0.079	0.096	0.028	0.056	0.144	0.205	0.076	0.124
platelets	0.194	0.212	0.112	0.123	0.101	0.106	0.042	0.050	0.181	0.195	0.098	0.109
cd40	0.190	0.237	0.108	0.147	0.183	0.234	0.102	0.186	0.187	0.223	0.103	0.135

a Average Jaccard value: the average Jaccard value of all clusters.

b Maximum Jaccard value: the Jaccard value of one cluster in which its Jaccard value is maximum in all clusters.

c Average PR value: the average PR value of all clusters.

d Maximum PR value: the PR value of one cluster in which its Jaccard value is maximum in all clusters.

### Assessment of the Clustering Performance on Protein Data

For protein data, the protein families are detected by the clustering algorithm. The Recall and Precision Measure Score (F-Measure Score) is used to measure the clustering results. F-measure score considers both recall score and precision score. For a cluster 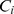 and a protein family 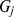, the precision 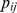 is the number of correct results divided by the cluster size (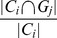) and the recall 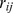 is the number of correct results divided by the protein family size (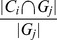). The F-measure score of 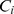 and 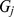 is calculated as 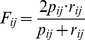.

**Figure 3:**
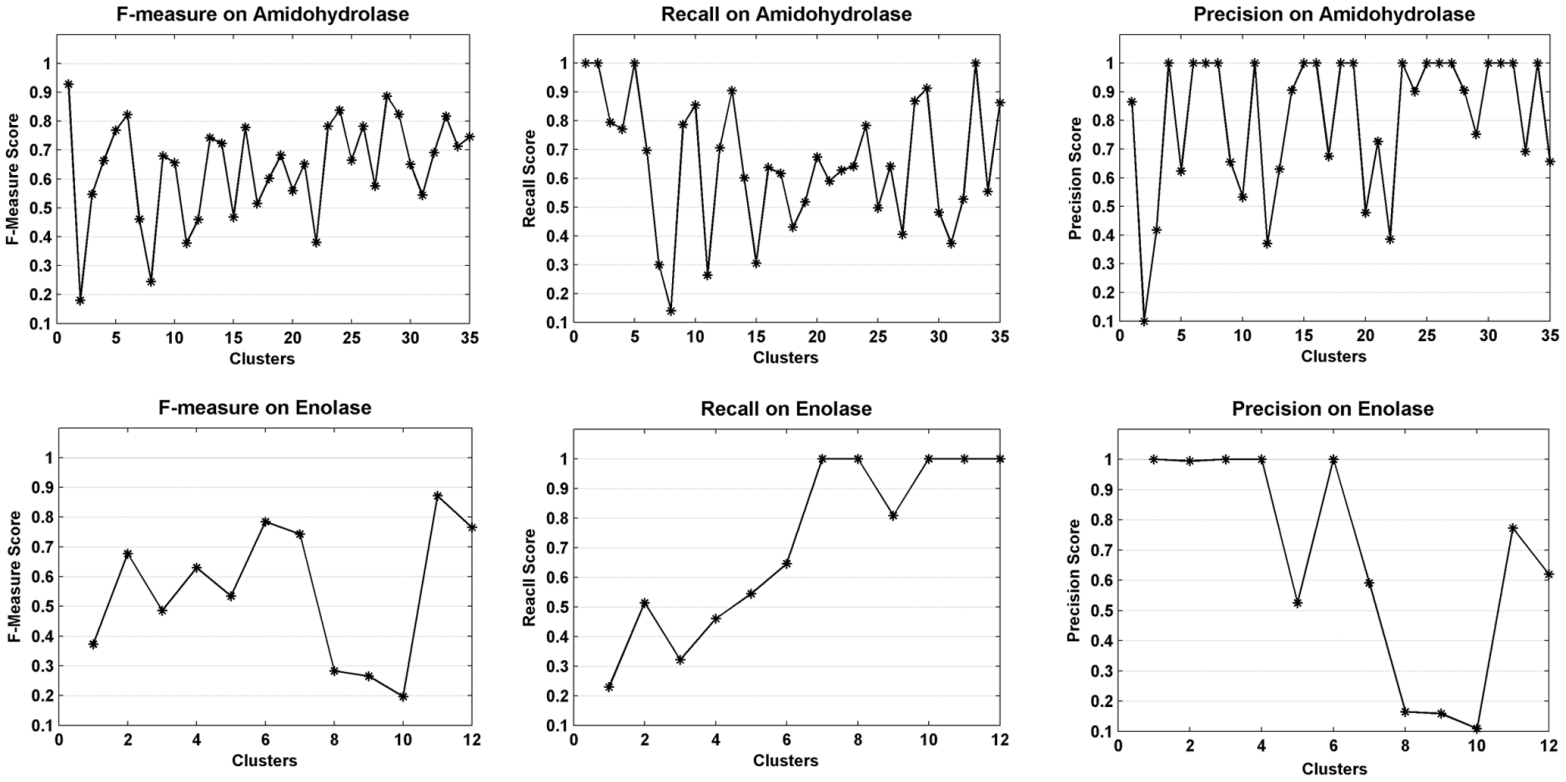
The F-Measure score of some large clusters on two protein data sets. The F-measure score, recall score and precision score are calculated. The 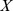 axis and 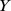 axis represent the cluster and its corresponding best score value, respectively. Some tiny and singleton clusters are not considered in the figure.

Two protein subsets with family annotations, containing 14927 and 5431 protein sequences, are regarded as the gold-standard and are clustered to measure the algorithm performance in the experiments. The F-measure score varies from 0 to 1, where 0 is the worst and 1 is the best. For each cluster, the maximum value is taken as the F-measure score over all protein families, and the average value over all clusters, weighted by cluster size, is taken as the F-measure score of a data set. The F-measure scores of two protein superfamilies are listed in Table 4, and Figure 3 shows the detailed recall and precision scores of some larger clusters (larger cluster means there are more than 10 data points in this cluster). The recall score means how many proteins in this family are detected by the AP algorithm in a gold-standard protein family; the precise score means how many proteins in this family are divided right. As shown in Figure 3, the x-axis represents the cluster number. For instance, 1 in x-axis means that the 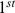 larger cluster; 2 in x-axis means that the 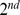 larger cluster. The AP detects about 35 larger protein families and about 12 larger protein families for the Amidohydrolase and Enolase protein superfamily, respectively. The results show that the algorithm can detect the protein families in the large protein superfamilies effectively. Most clusters achieve a high precision score. Both precision and recall scores indicate that most proteins in the same families are detected and grouped correctly.

**Table 4:** Clustering performance on protein superfamily data sets.

Data Sets	Amidohydrolase	Enolase
Superfamily Size	14927	5431
Family Number	80	19
Cluster Number	85	20
F-Measure Score	0.57	0.54

### Error Rates Analysis of Parallel and Non-Parallel Algorithms

In order to validate that the parallel affinity propagation algorithm performs well and its results are similar to the non-parallel affinity propagation algorithm, we make the error rates analysis of both algorithms. We get the non-parallel program and a relatively small data set (“Facesimilarity” data set, 900 data points) from the original publication of AP algorithm [21], and then run this non-parallel program and our parallel program on this data set. According to its description, it is appropriate to set the “preference value” (input of AP algorithm) close to median of the similarity values. So we set a range of the “preference value” which is close to the median of the similarity values, and use these “preference values” to test both algorithms. For all input values, both algorithms detect the same number of clusters, and converge at the same iteration steps. Furthermore, because the cluster structure is extracted from the latest message matrices, it is important to validate whether the two message matrices of both algorithms are the same. We calculate the 2-norm of the difference value of two message matrices got from both parallel and non-parallel algorithms. Detailed results are listed in Table 5.

**Table 5:** Error rates analysis of parallel and non-parallel algorithms.

	Convergence Steps^a^		Cluster Num^b^		Error Rates^c^	
Preference^d^	Non-P^e^	P^f^	Non-P	P	R msg^g^	A msg^h^
−40	123	123	94	94	1.2703e-04	1.7147e-04
−50	118	118	72	72	1.2967e-04	2.0939e-04
−60	129	129	62	62	1.2364e-04	1.9748e-04
−70	112	112	50	50	1.4744e-04	2.6443e-04
−80	117	117	46	46	1.1632e-04	2.4990e-04

a Convergence steps: the iteration steps when the algorithm is converged.

b Cluster Number: the number of clusters which the AP algorithm detects.

c Errot rates: the 2-norm of the difference value of message matrices from two algorithms.

d Preference value: the input value of AP algorithm.

e Non-Parallel algorithm: the non-parallel version of algorithm which get from the original publication of AP algorithm.

f Parallel algorithm: the parallel version of algorithm.

g Responsibility message: the responsibility message of AP algorithm.

h Availability message: the availability message of AP algorithm.

### Conclusion

The parallel affinity propagation clustering algorithm can address the large-scale biological problems such as clustering the microarrays gene data sets and detecting the protein families effectively. The Euclidean distance and BLAST E-value are used in the experiments to describe the similarities of gene data pairs and protein data pairs, respectively. In the similarity matrix construction, the memory-shared architecture minimizes the communication time between data pairs, and the shutter partition of data sets balances the computing loads on cores. Compared with the sequential algorithm, the parallel affinity propagation algorithm reduces the runtime significantly and achieves promising speedups. Also, it handles large-scale biological data sets effectively. From the GO overlap score and F-measure score, the affinity propagation is validated so that it is very feasible in clustering the gene data sets and detecting the protein families. Besides the analysis of microarray gene data and protein families, it is possible and promising to apply the affinity propagation algorithm into some other researches like microbial communities.

## Discussion

### Appropriate Parallel Architecture

There are two steps for affinity propagation algorithm to cluster a data set. The first one is constructing the similarity matrix, and the second is exchanging messages (responsibility message and availability message). However, considering the strong correlations between each data points in the procedure of constructing similarity matrix, we implemented parallel similarity matrix construction algorithm in the memory-shared architecture. The parallelism in this kind of parallel architecture is on the thread level, and threads share the memory with each other, so there are nearly no communication cost among threads. For the affinity propagation algorithm, it always requires large memory space to store intermediate information when clustering large-scale data sets. Since the distributed system consisting of many computers, large memory size and great computing capacity are available, it become the best choice for the parallel affinity propagation algorithm.

### Minimizing Global Communication

The increase of communication cost leads to a longer algorithm runtime and a lower speedup. In all kinds of communication, the global communication is the most time consuming. The affinity propagation algorithm is parallelized on the process-level, and each core can run one or several processes concurrently. According to two message computing rules, nearly 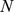 times global communication are require in each iteration to gather information from all processes. In order to reduce the global communication cost in each iteration, the similarity matrix is partitioned by the row and some variables are defined to store the intermediate results when computing the availability message. In this way, the responsibility message is computed independently without communication among processes; for the availability message, it requires only one time global communication in each iteration. In our methods, the global communication is minimized to 1 time in one iteration, so the communication cost is reduced significantly.

## Materials and Methods

### Parallel Constructing Similarity Matrix

**Figure 4:**
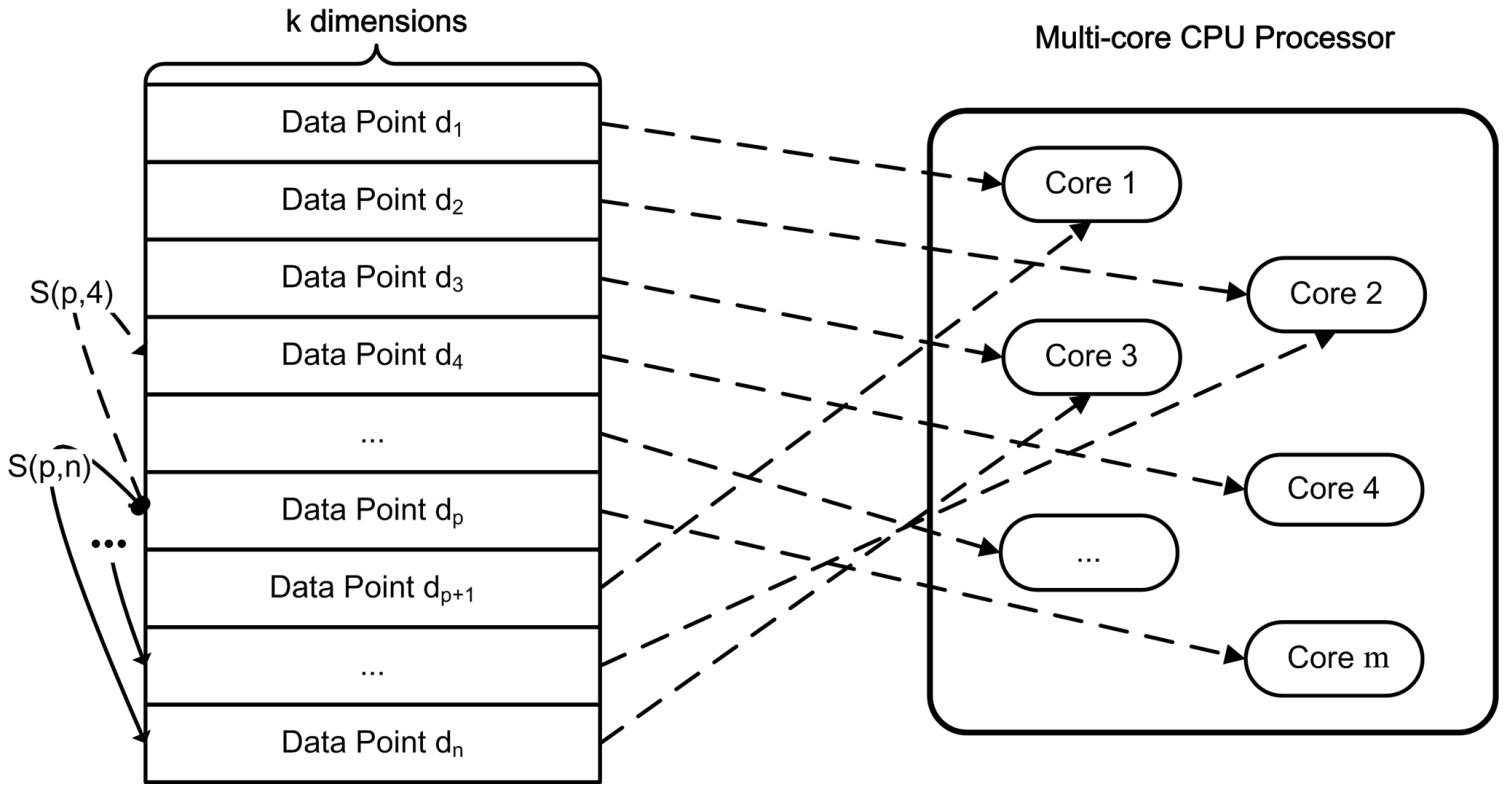
Partition of the input biological matrix. Rows of the input matrix are assigned to different cores to calculate the similarities with others (each row represents a data point; 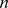 and 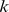 represent the row number of the input matrix and the dimension of each row, respectively; 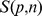 represents the similarity between data point 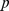 and 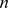; 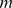 represents the number of available cores).

Since affinity propagation algorithm runs based on the similarities between data pairs, the similarity matrix is required to be constructed firstly. There exist many methods to construct the similarities matrix, including the Euclidean distance, Pearson correlation coefficient, E-value(output of BLAST software) and so on. The time complexity of constructing the similarity matrix is about 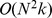, where 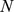 and 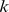 represent the data point number and the dimension of each data points, respectively. The biological data sets are partitioned and several cores are used to accelerate the construction procedure of similarity matrix. Figure 4 presents the partition of data sets.

The shutter partition way rather than the sequence way, is used to to alleviate the unbalanced computing load on cores. The sequential partition means that rows of the input matrix are assigned to cores one by one; while the shutter partition means that rows of the input matrix assigned to cores one block by one block, and the length of block equals the number of available cores. For instance, given an input matrix, with 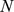 rows, the number of available cores is 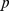. All cores, except for the last one, are assigned about 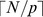 rows (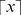 means round up×to an integer). For the sequential partition, the 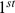, 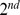, 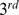,…, 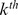 rows are assigned to the 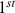 core (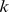 equals 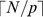); 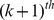, 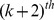,…, 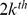 rows are assigned to the 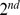 core, and so on. For the shutter partition, the length of block equals 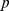 (number of available cores), and the 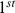, 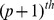, 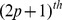,…, 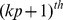 rows are assigned to the 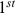 core; the 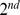, 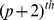, 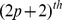,…, 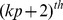 rows are assigned to the 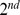 core, and so on.

**Figure 5:**
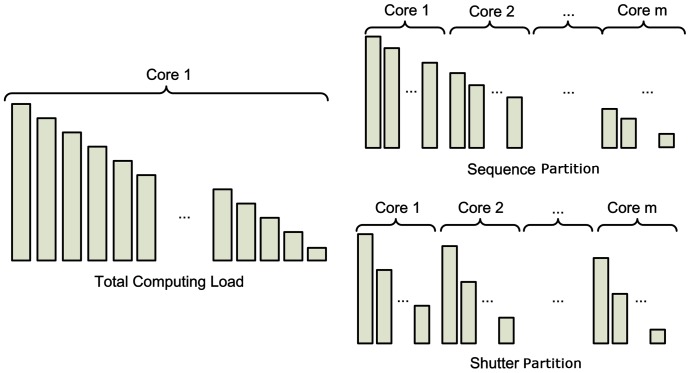
The computing load on cores for different data partition ways. (a) The computing load of whole data set on one core. The computing loads on the cores which are assigned the upper rows of input matrix are much more than that on the cores which are assigned the lower rows. (b) The computing load on cores when the input matrix is partitioned by the sequence partition. (c) The computing load on cores when the input matrix is partitioned by the shutter partition.

In the experiments, the Euclidean distance is taken to describe the similarities between data points. As shown in Figure 4, data point 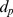 is required to compute the similarities with data points 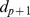, 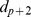,…, 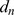 and doesn't require to compute with 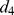. Because the value of 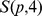 equals 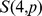, and the value has been computed previously. The symmetry of calculating similarity leads to the unbalanced computing load of the rows of input matrix. The rows at the bottom of input matrix require less calculation than those at the top of input matrix. So for the sequential partition, the computing loads on the cores, which are assigned the upper rows of input matrix, are much more than that on the cores, which are assigned the lower rows. It causes unbalanced computing loads on cores. However, for the shutter partition, cores are assigned rows of the whole input matrix, so the computing load is much more balanced. It makes the cores fully utilized. Figure 5 depicts computing load on cores by two partition ways.

### Parallel Affinity Propagation Algorithm

The affinity propagation algorithm clusters data set based on the similarity matrix, and exchanges two messages between data points. The two messages are responsibility and availability messages. The responsibility 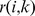 and availability messages a(i,k) are computed by the following rules.
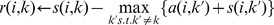
(1)

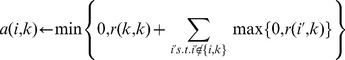
(2)


When the algorithm runs, three information matrices (one for similarity and two for messages) are required to be stored in the memory. In the information matrices, each row or column represents one data point. For large-scale data sets, it is impossible for the stand-alone computer to run the sequential affinity propagation algorithm because of the nearly endless runtime and the limitation of memory space. Given a data set with more than 50000 data points, it requires nearly 56 GB (double precision) memory space to store three information matrices and the algorithm runtime is more than 30 hours. The parallel affinity propagation in the distributed system can alleviate this kind of limitation significantly.

**Figure 6:**
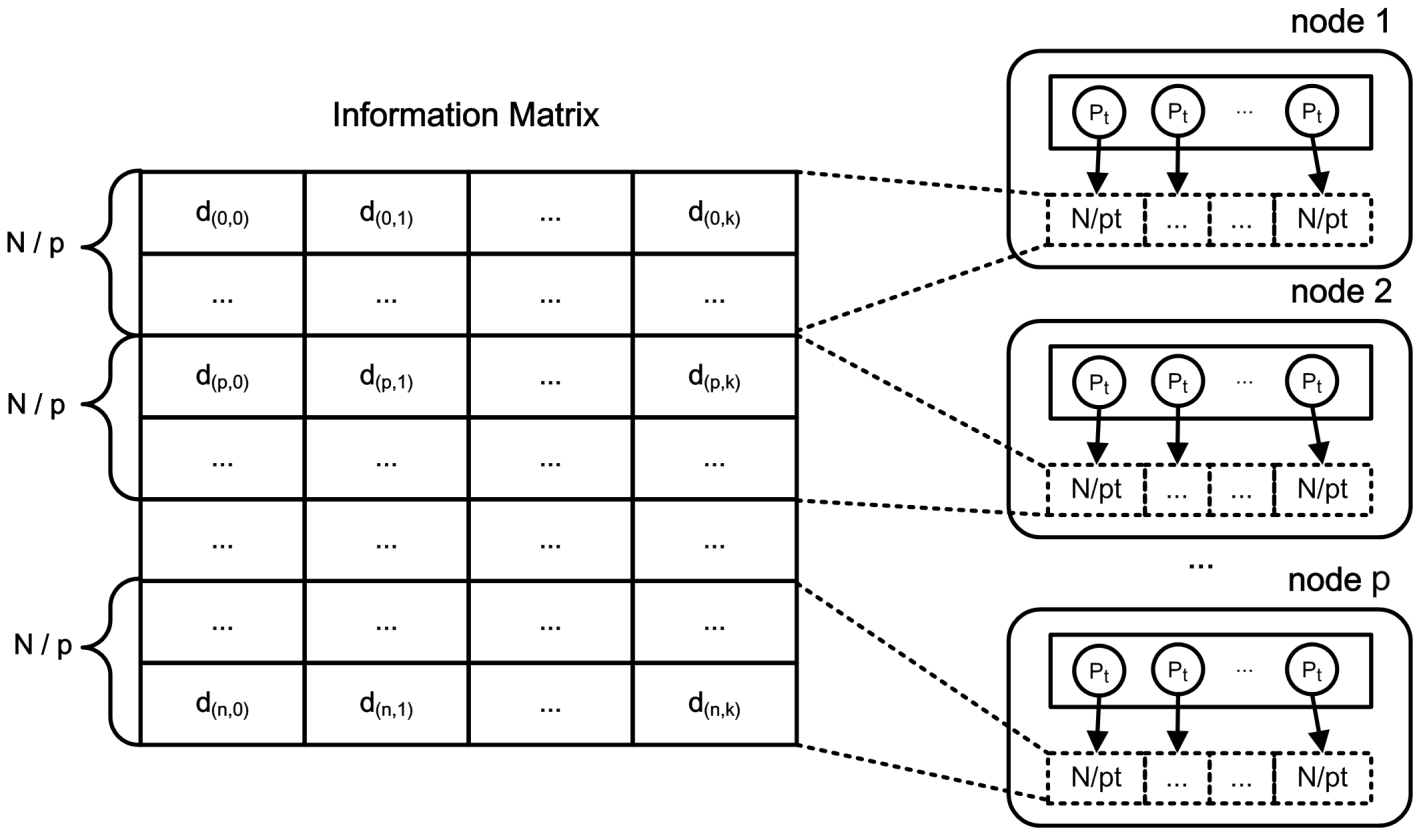
Partition of three information matrices. All three matrices are partitioned by the row. For a matrix with 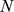 rows and a computing cluster with 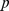 machine nodes, each node is assigned about 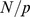 rows of each information matrix. In each node, the 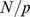 rows are processed by 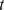 cores concurrently.

In the distributed system, affinity propagation algorithm runs on the process-level parallelism. Each process is assigned a few rows of the three information matrices, and each core execute one or several processes concurrently. From the equation 1 and equation 2, we know that the responsibility message is calculated by the row, and the availability message is calculated by the column. Compared with the responsibility message, the availability message has less correlations among data points. As shown in Figure 6, in order to reduce the communication cost in parallel algorithm, we partition the information matrices by the row.

The key issue in the parallel affinity propagation algorithm is to parallel exchange messages among data points. According to the message computing rules, the responsibility message is computed by the rows and the availability message is computed by the columns. Since the information matrices are partitioned by the rows, the data chunks of each row are all stored in the local running process when computing the responsibility message. So the responsibility message can be computed independently without communication cost among processes. For the availability message, the summation values of all columns in the information matrices are computed. However, since data chunks of each column are distributed on the different running processes, data chunks required to be gathered from all processes for 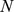 times to compute the summation values of 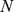 columns, causes a massive communication cost.

**Figure 7:**
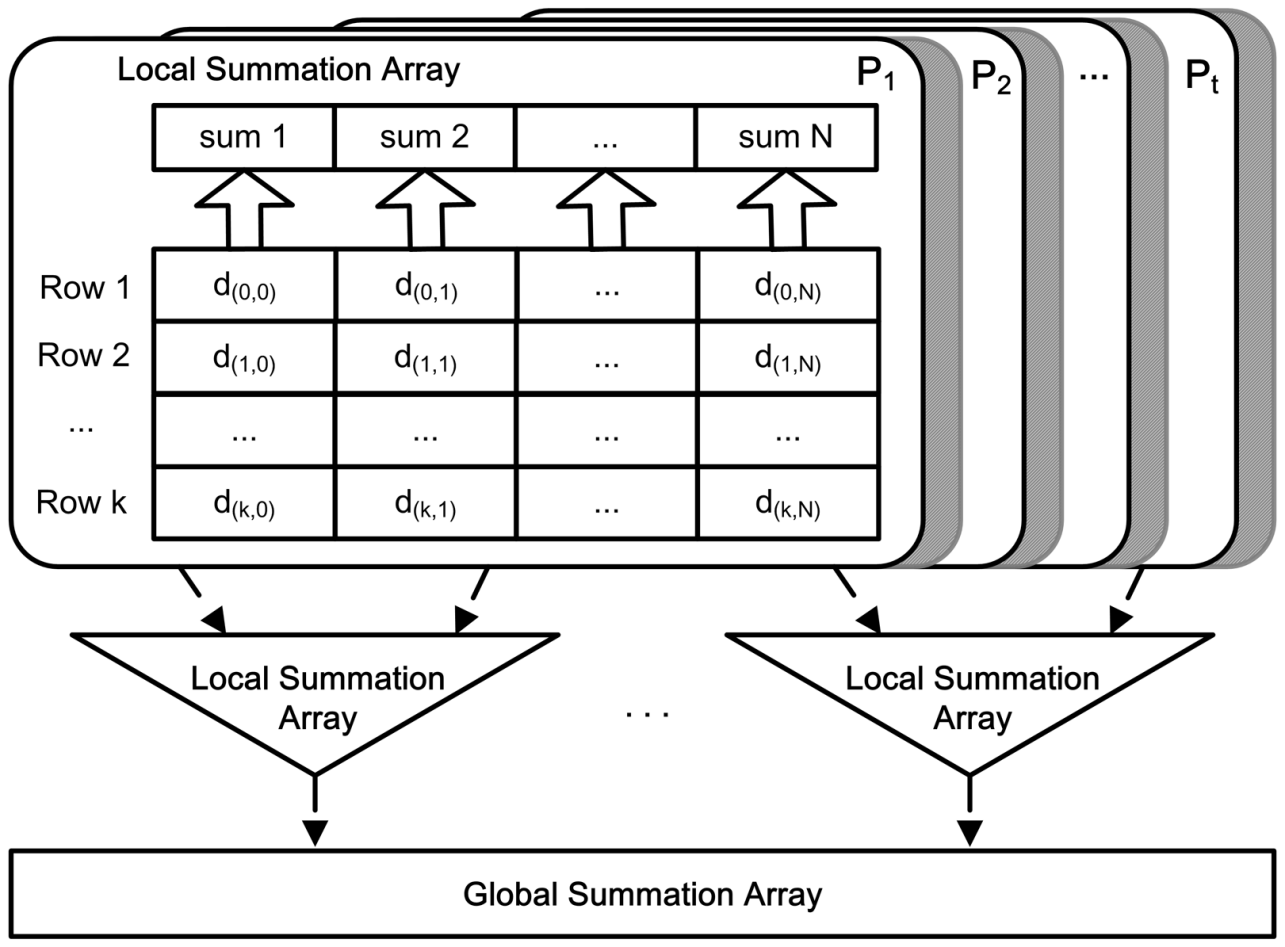
The procedure of computing availability messages. There are 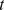 processes 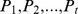, and each process is assigned about 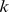 rows of the availability messages matrix after partitioning. In each row, there are 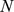 column. In order to reduce the communication cost, each process computes the local summation of the 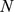 columns and stores the intermediate values in the Local Summation Array firstly, and then these local values in all processes are gathered and scattered to compute the global summation of the 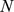 columns.

In order to minimize the global communication times, the local summation of 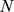 columns is computed firstly in each process, and intermediate variables are defined to store these intermediate results. In this way, the root process requires only one time global reduction and scatter to finish computing availability message. The communication cost is optimized from 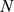 times global communication to 1 time. Figure 7 depicts the procedure of parallel computing availability message, as well as the pseudocode of parallel computing availability message listed in the Table 6.

**Table 6:** Algorithm 1.

1: **function** avaimsgcpt(*Res*, *Avai*, *lam*)
2: **for** *i*<*colSize* **do**
3: **for all** *v* ∈ *Res*[*i*] **do**
4: *localsum*[*i*]←*localsum*[*i*]+*max*(0, *v*)
5: **end for**
6: **end for**
7: *globalsum*←*MPI_Allreduce*(*localsum*)
8: *MPI_Barrier* ()
9: **for** *i*<*colSize* **do**
10: **for** *k*<*rowSize* **do**
11: *v*←*max*(0, *Res*[*k*][*i*])
12: *A*[*k*][*i*]←*min*(0, *globalsum*[*i*]−*v*)
13: **end for**
14: **end for**
15: *Avai*←*A*×(1−*lam*)+*Avai*×*lam*
16: **end function**

Availability message computation.

### Data Sets

Two kinds of data sets are used here, including the gene data sets and protein data sets. DNA microarrays are extremely powerful tools for such studies in which they allow one to probe virtually the entire transcriptome to give an overall picture of gene expression behavior [35]. Microarray data sets are commonly very large, and are influenced by a number of variables. Many clustering algorithms currently have been introduced to explore genes expression data [36]–[38]. Three microarray data sets are used in the experiments, including TSEMFC (Tobacco smoke effect on maternal and fetal cells, GEO Accession Number: GSE27272) [39], platelets (platelets sickle cell disease, GEO Accession Number: GSE11524) [40] and cd40 (CD40-Ligand screen in B cells, GEO Accession Number: GSE376) [41] which are downloaded from the Gene Expression Omnibus online database (GEO) [42]. TSEMFC data set comes from the analysis data of peripheral blood leukocytes and placenta of pregnant smokers, with 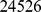 data points and 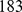 samples. The platelets data set comes from the analysis data of platelets from patients with sickle cell disease, with 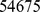 data points and 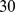 samples. The cd40 data set is the data of effects induced by 65 nM CD40 in male c57BL/6 B cells and consists of 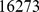 data points and 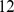 samples.

For protein data sets, protein families are detected in the superfamily by the affinity propagation algorithm. Detecting protein families in large-scale superfamily is a difficult but important biological task. Proteins with the same functions or biological processes should be in the same protein families [43]. Moreover, the protein families are always defined as the groups of molecules which share significant sequence similarities [44], so it is promising to detect the protein families through the protein sequence similarities. Similarity matrices of protein data sets are constructed by carrying out the all-against-all BLAST in local databases, and the similarities between protein pairs are set to the 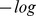 of the BLAST E-value. Two large protein superfamilies are downloaded from Structure-Function Linkage database [45]. They are Amidohydrolase [46] and Enolase [47] protein superfamilies, with about 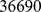 and 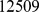 protein sequences, respectively.
